# Electrocardiogram artifact caused by rigors mimicking narrow complex tachycardia: a case report

**DOI:** 10.1186/1756-0500-7-80

**Published:** 2014-02-04

**Authors:** Anne Thushara Matthias, Jegarajah Indrakumar

**Affiliations:** 1 University Medical Unit, Colombo South Teaching Hospital, Colombo, Sri Lanka; 2 Department of Medicine, University of Sri Jayewardenepura, Nugegoda, Sri Lanka

**Keywords:** Arrhythmia, Supra ventricular tachycardia, Rigors

## Abstract

**Background:**

The electrocardiogram (ECG) is useful in the diagnosis of cardiac and non-cardiac conditions. Rigors due to shivering can cause electrocardiogram artifacts mimicking various cardiac rhythm abnormalities.

**Case presentation:**

We describe an 80-year-old Sri Lankan man with an abnormal electrocardiogram mimicking narrow complex tachycardia during the immediate post-operative period. Electrocardiogram changes caused by muscle tremor during rigors could mimic a narrow complex tachycardia.

**Conclusions:**

Identification of muscle tremor as a cause of electrocardiogram artifact can avoid unnecessary pharmacological and non-pharmacological intervention to prevent arrhythmias.

## Background

The electrocardiogram (ECG) is an important bedside investigation technique used for the diagnosis of cardiac diseases. ECG rhythm abnormalities can also occur in many non-cardiac conditions mimicking cardiac pathology, including activity of muscles. The possibility of an artifact causing ECG findings should be considered in an otherwise asymptomatic patient who is haemodynamically stable. This case report describes a patient who had rigors during the post-operative period and developed ECG changes that were initially thought to be an arrhythmia. The ECG changes reflected artifacts caused by activity of muscles.

## Case presentation

**Figure 1:**
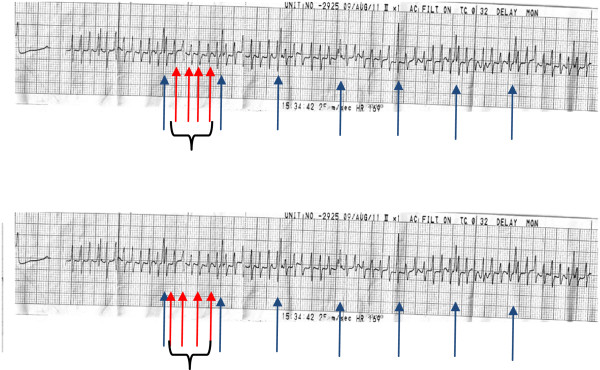
**Electrocardiogram taken during the rigors.** Blue arrow: normal QRS complexes. Red arrows bracketed by black brackets: Noise – artifact.

We report a case of an 80-year-old man who underwent a hemiarthroplasty. He had no drug or food allergies. He was admitted to the hospital after an accidental fall and an x-ray showed fracture of the neck of the left femur. Following surgery under spinal anaesthesia, he was brought to the Intensive Care Unit (ICU) for routine observation. The patient was conscious and rational. On arrival to the ICU, the medical officer noticed that the patient was shivering, and covered him with a blanket. He had no other complaints. The patient’s temperature was 35.8°C. The Glasgow coma scale was 15. The pulse rate felt was 92 beats per minute, the monitor reading was 220 beats per minute, and the blood pressure was 100/60 mmHg. Cardiac auscultation was difficult because of shivering. Auscultation revealed vesicular breathing with no added sounds in the lungs. The examination of the abdomen was normal. The junior medical officer noticed an abnormal rhythm shown in the cardiac monitor, and informed the consultant physician. An ECG rhythm strip taken at the time is shown in Figure 1.

Preoperative assessment of liver function tests, blood urea, serum creatinine, serum sodium, serum potassium, erythrocyte sedimentation rate, prothrombrin time and international normalized ratio, thyroid function, haemoglobin, and platelet count were all within normal range. Acting on the ECG changes, the medical officer performed a carotid sinus massage, and then administered adenosine. However, there was no change in the ECG. The patient was cold to touch, and temperature was recorded as 34.9°C. The patient was then covered to prevent heat loss. The ECG changes then gradually resolved, and ECG was normal when repeated 30 min later.

## Discussion

An ECG artifact is an abnormality that is not due to the electrical activity of the heart [1]. Failure to identify artifacts can lead to incorrect diagnoses and mismanagement of patients. Tremor or shivering was previously reported to be common during the postoperative period in a hypothermic patient [2]. Our patient developed shivering that caused an artifact in the ECG that mimicked a narrow complex tachycardia, which alerted the medical officer to urgently contact the physician.

When skeletal muscles undergo tremors, the electrical activity can cause the ECG to develop an irregular baseline. These muscle tremors are known to frequently cause ECG abnormalities that may mimic cardiac pathology, including ventricular tachycardia [3] and atrial flutter [4,5]. In our case, the presence of normal QRS complexes (blue arrow) in the midst of the narrow complexes (red arrow) indicated that muscle tremor caused an irregular ECG baseline. On first appearance, the ECG mimicked a narrow complex tachycardia. However, close observation showed normal QRS complexes among the irregular baseline, with a rate of approximately 60 beats per minute. A number of reports have described the presence of the “Notch Sign” [6]. In our case, the “Notch Sign” indicated the presence of normal QRS complexes, which were visible in the electrocardiographic artifact amidst the narrow complexes, at intervals that coincided with the cycle length of the baseline rhythm of the patient and the post shivering rhythm. Thus, the “Notch Sign” was an important sign to look for in an ECG tracing and the diagnosis of the ECG was due to muscle tremor causing an ECG artifact.

Other characteristics that aided in differentiating this artifact from supra ventricular tachycardia included the absence of hemodynamic deterioration during the episode, the patient was asymptomatic, and the presence of rigors. Correlation of the patient’s clinical parameters such as blood pressure, heart rate on palpation, and saturation on the pulse oximeter also helped the junior doctors in making the diagnosis of an artifact. Thus, it is important to consider muscle tremor as potential common cause of ECG artifacts in the postoperative stage to avoid making erroneous diagnoses, especially by a junior doctor on duty in the ICU. In the present case, if the junior doctor had noticed that the heart rate was quite low compared to that expected in supra ventricular tachycardia, it could have prevented the administration of adenosine. A 12-lead ECG and close attention to the patient, rather than just the ECG on the monitor, could have prevented the erroneous diagnosis.

## Conclusion

ECG artifacts caused by muscle activity can mimic a supraventricular tachyarrhythmia. Methods to eliminate rigors such as keeping the patient warm can eliminate these artifacts. This report emphasized the clinical importance of correlating the patient’s clinical condition in the appropriate environment with the ECG changes. This will avoid unnecessary anxiety and even emergency treatment by less experienced medical officers in the ICU.

## Consent

Written informed consent was obtained from the patient for publication of this case report and accompanying images. A copy of the written consent is available for review by the Editor-in-Chief of this journal.

## Competing interests

The authors declare that they have no competing interests.

## Authors’ contributions

Both authors contributed equally. Both authors read and approved the final manuscript.

## Authors’ information

Anne Thushara Matthias (MBBS), Registrar in Medicine, University Medical Unit, Colombo South Teaching Hospital.

Professor Jegarajah Indrakumar (MBBS, MD, FRCP), Professor in Medicine, Department of Medicine, University of Sri Jayewardenepura.
